# Supplementary material for: Modelling and Optimisation of Multi-Stage Flash Distillation and Reverse Osmosis for Desalination of Saline Process Wastewater Sources
Source: Membranes (Basel). 2020 Sep 28;10(10):265. doi: 10.3390/membranes10100265 (PMC7600958; doi:10.3390/membranes10100265)
Supplement: Supplementary file 1 [file membranes-10-00265-s001.pdf]

*Supplementary Materials*

# Modelling and Optimisation of Multi-Stage Flash Distillation and Reverse Osmosis for Desalination of Saline Process Wastewater Sources

**Andras Jozsef Toth** [1, 2*]

[1] Environmental and Process Engineering Research Group, Department of Chemical and Environmental Process Engineering, Budapest University of Technology and Economics, H-1111, Hungary, Budapest, Műegyetem rkp. 3.

[2] Institute of Chemistry, University of Miskolc, Egyetemváros C/1 108, H-3515, Miskolc, Hungary

* Correspondence: andrasjozseftoth@edu.bme.hu; Tel.: +36-1-463-1490; Fax: +36 1 463 3197, ORCID: 0000-0002-5787-8557

**Table S1.** Comparison of industrial and simulated data: Temperature of recirculating brine entering each flash stage.

| Temp. of Recirculating Brine Entering Each Flash Stage [°C] | | | |
|---|---|---|---|
| **Stage Number** | **Industrial Data** | **Simulated Data** | **Error [%]** |
| 1 | 83.2 | 82.8 | -0.5 |
| 2 | 81.1 | 80.7 | -0.5 |
| 3 | 78.8 | 78.5 | -0.4 |
| 4 | 76.4 | 76.3 | -0.1 |
| 5 | 74.0 | 73.7 | -0.4 |
| 6 | 71.7 | 71.4 | -0.4 |
| 7 | 69.4 | 69.2 | -0.3 |
| 8 | 67.1 | 66.9 | -0.3 |
| 9 | 64.8 | 64.4 | -0.6 |
| 10 | 62.7 | 62.4 | -0.5 |
| 11 | 60.5 | 60.3 | -0.3 |
| 12 | 58.3 | 58.2 | -0.2 |
| 13 | 56.2 | 56.0 | -0.4 |
| 14 | 54.1 | 53.9 | -0.4 |
| 15 | 52.0 | 51.8 | -0.4 |
| 16 | 49.9 | 49.8 | -0.2 |
| 17 | 47.9 | 47.7 | -0.4 |
| 18 | 45.9 | 45.8 | -0.2 |
| 19 | 44.0 | 43.7 | -0.7 |
| 20 | 42.1 | 42.0 | -0.2 |
| 21 | 40.3 | 40.2 | -0.2 |
| 22 | 38.0 | 37.9 | -0.3 |
| 23 | 35.3 | 35.2 | -0.3 |
| 24 | 32.2 | 32.2 | 0.0 |

**Table S2** Comparison of industrial and simulated data: Distillate produced from each stage

| Distillate Produced From Each Stage [Ton/min] | | | |
|---|---|---|---|
| **Stage Number** | **Industrial Data** | **Simulated Data** | **Error [%]** |
| 1 | 0.690 | 0.692 | 0.24 |
| 2 | 0.870 | 0.872 | 0.19 |
| 3 | 0.930 | 0.933 | 0.36 |
| 4 | 0.950 | 0.950 | 0.00 |
| 5 | 0.940 | 0.942 | 0.18 |
| 6 | 0.910 | 0.917 | 0.73 |
| 7 | 0.880 | 0.883 | 0.38 |
| 8 | 0.860 | 0.867 | 0.77 |
| 9 | 0.850 | 0.853 | 0.39 |
| 10 | 0.830 | 0.837 | 0.80 |
| 11 | 0.820 | 0.827 | 0.81 |
| 12 | 0.810 | 0.815 | 0.61 |
| 13 | 0.800 | 0.805 | 0.62 |
| 14 | 0.780 | 0.785 | 0.64 |
| 15 | 0.770 | 0.772 | 0.22 |
| 16 | 0.750 | 0.750 | 0.00 |
| 17 | 0.730 | 0.733 | 0.45 |
| 18 | 0.720 | 0.722 | 0.23 |
| 19 | 0.700 | 0.700 | 0.00 |
| 20 | 0.680 | 0.683 | 0.49 |
| 21 | 0.650 | 0.650 | 0.00 |
| 22 | 0.550 | 0.550 | 0.00 |
| 23 | 0.630 | 0.633 | 0.53 |
| 24 | 0.700 | 0.700 | 0.00 |

**Table S3** Comparison of industrial and simulated data: Outlet pressure from each stage

| Outlet Pressure From Each Stage [bar] | | | |
|---|---|---|---|
| **Stage Number** | **Industrial Data** | **Simulated Data** | **Error [%]** |
| 1 | 0.638 | 0.640 | 0.31 |
| 2 | 0.585 | 0.590 | 0.85 |
| 3 | 0.536 | 0.540 | 0.74 |
| 4 | 0.488 | 0.490 | 0.41 |
| 5 | 0.436 | 0.440 | 0.91 |
| 6 | 0.398 | 0.400 | 0.50 |
| 7 | 0.368 | 0.370 | 0.54 |
| 8 | 0.327 | 0.330 | 0.91 |
| 9 | 0.298 | 0.300 | 0.67 |
| 10 | 0.278 | 0.280 | 0.71 |
| 11 | 0.248 | 0.250 | 0.80 |
| 12 | 0.228 | 0.230 | 0.87 |
| 13 | 0.209 | 0.210 | 0.48 |
| 14 | 0.189 | 0.190 | 0.53 |
| 15 | 0.169 | 0.170 | 0.59 |
| 16 | 0.149 | 0.150 | 0.67 |
| 17 | 0.139 | 0.140 | 0.71 |
| 18 | 0.119 | 0.120 | 0.83 |
| 19 | 0.109 | 0.110 | 0.91 |
| 20 | 0.100 | 0.100 | 0.10 |
| 21 | 0.090 | 0.090 | 0.33 |
| 22 | 0.090 | 0.090 | 0.56 |
| 23 | 0.080 | 0.080 | 0.19 |
| 24 | 0.070 | 0.070 | 0.10 |
